# Neural dysfunctions associated with atypical memory in ASD: an ALE meta-analysis

**DOI:** 10.3389/fpsyt.2026.1830424

**Published:** 2026-08-21

**Authors:** Lang Chen, Meghan Abate, Mackenzie Fredericks, Yuanchun Guo, Zhizhen Tao

**Affiliations:** 1 Department of Psychology, Santa Clara University, Santa Clara, CA, United States; 2 Neuroscience Program, Santa Clara University, Santa Clara, CA, United States; 3 School of Education and Counseling Psychology, Santa Clara University, Santa Clara, CA, United States

**Keywords:** ALE meta-analysis, autism, fronto-parietal network, hemispheric asymmetry, memory

## Abstract

Autism Spectrum Disorder (ASD) is now one of the most prevalent neurodevelopmental disorders, characterized by socio-communicative and cognitive challenges. Amongst them, memory challenges in ASD have been reported early, but only recently have studies suggested a prevalent memory impairment in autistic individuals. More importantly, little is known about the brain correlates associated with the memory challenges in ASD. In the current study, we used both quantitative and qualitative approaches to review a total of 42 studies (22 were included in the ALE analysis) to reveal the consistent findings in the literature on the memory functions in ASD, and found that the memory challenges in ASD are probably accompanied by an imbalance between brain functions in the left and right hemispheres, with deficient functions in fronto-parietal regions on the left hemisphere and an excessive engagement of the counterparts on the right hemisphere. In addition, the analysis suggested a top-down deficit from the frontal lobe during the memory tasks, and the atypical functions probably depended on the stimulus format and task types.

## Introduction

Autism Spectrum disorder (ASD) is a prevalent neurodevelopmental disorder, known for its characteristic symptoms in socio-communicative skills and repetitive, restricted interests and behaviors (1–3). Surprisingly, although the early reports of ASD have suggested a memory deficit in individuals with ASD, their challenges in learning and memory were only highlighted recently (4–7). Furthermore, little is known about the neurobiological basis of their memory dysfunction due to limited research (8, 9), especially since no quantitative analysis was done to examine the convergence of existing findings. Thus, the current study aimed to reveal the consistent findings of neural dysfunctions associated with atypical memory ability in ASD using a meta-analytical approach.

Memory is an important cognitive component for every aspect of our daily functioning, and early studies have focused on memory challenges relevant to the social and verbal abilities of ASD (10, 11). For example, face recognition is considered a core aspect of social ability (12, 13), and is relatively well-studied in ASD (14, 15). Since 1943, studies have proposed that autistic individuals have a hard time differentiating between faces (16), and later research reported that children later diagnosed with ASD tended to avoid eye areas when looking at people and challenges in matching names to faces (17–20). More recent work (21) revealed that autistic individuals scored significantly lower on various face memory tasks and also reported increased difficulty with face recognition compared to non-autistic individuals.

Face memory challenges in ASD are conventionally regarded as an outcome of a domain-specific impairment in social functions (22), but recent work also suggests that face memory challenges in ASD originate from a domain-general dysfunction in memory representations (6, 23–25). Indeed, other studies using non-facial visual stimuli to examine the memory functions in ASD also revealed noticeable impairment. For instance, in a visual object memory retrieval task, autistic individuals showed a higher error rate and less precise responses (26). Studies have also suggested that their visual memory challenges may extend to visuospatial information (27, 28). Therefore, evidence suggests that the visual memory challenges in ASD may not be confined to socially salient objects like faces, but also to other visual stimuli (23, 25, 29, 30).

Since communication and language have also been described as hallmark deficits in autistic individuals (31, 32), existing research also emphasizes the impaired verbal memory in ASD (11, 33). For instance, autistic individuals performed worse on standardized verbal memory tests than their age- and IQ-matched non-ASD group (34). Autistic individuals were shown to have difficulty in holding verbal information for a short term (35), free-recalling words, and discriminating between old and new words (36). Their verbal memory challenges relate to their adaptive behaviors (37), task complexity (38), and cognitive load (39). Thus, there is a consensus that autistic individuals are likely to have memory challenges for verbal and visual stimuli.

Besides the memory format(s), namely, visual vs. verbal, previous behavioral studies examined the different types of memory in ASD. Regarding working memory (WM), which requires holding and manipulating information within a short time span (40, 41), previous research indicates that autistic individuals preserve an intact ability for simple tasks (e.g., 1-back). However, as the cognitive load increases (e.g., 2-back), autistic individuals show lower performance compared to non-ASD controls (6, 39). Studies also suggested that spatial working memory is particularly challenging for ASD (11, 28, 39). Thus, while considered a fundamental aspect of domain-general executive functions, working memory may play a pivotal role in the real-time processing of complex cognitive information during social cognition and interpersonal interactions (42, 43). For long-term memory, studies generally found that autistic individuals showed comparable ability for semantic memory, but more challenges for episodic memory (7, 24, 44, 45; but also see, 46). For instance, previous literature revealed that autistic adults show diminished abilities in both encoding and retrieval of associative information (36, 47). Meta-analytic studies have demonstrated that episodic memory is generally reduced in ASD with a small to medium effect size, albeit smaller than the effect size for short-term/working memory (24, 30). However, recent research points to weak representation in ASD for their episodic memory challenges (48–51) and revealed subtypes of atypical memory representations (23).

Although limited, neuroimaging studies have examined the neural correlates associated with memory challenges in ASD, and varied in terms of the format(s) of stimuli used (visual vs. verbal) as well as the types of memory (WM vs. episodic memory). A very recent review has comprehensively and qualitatively examined existing neuroimaging studies on memory functions in ASD (9). The general conclusion was that autistic individuals seem to rely more on the left hemisphere, posterior, and ventral aspects of the brain during memory tasks compared to non-ASD groups, suggesting excessive engagement of atypical regions for memory functions. The influence of the stimulus format (visual vs. verbal) remains unclear. For visual memory, the fusiform face area (FFA) is commonly examined for faces in ASD, and the majority of the work showed insufficient activation in the right FFA (52). Other visual memory tasks also seem to involve the ventral temporal cortex as well as the lateral occipital cortex and lingual gyrus (53–55). Conversely, the studies focusing on verbal memory revealed group differences in prefrontal regions during working memory tasks (39, 56), and inferior frontal gyrus, hippocampus, and angular gyrus (36, 57) between ASD and non-ASD groups. In terms of types of memory, the results were inconsistent. The findings on working memory were more mixed. For example, one study reported significant ASD vs. non-ASD group differences in frontal-parietal regions (58), but the other study found no significant group differences in temporo-occipital areas (39). The hippocampus was mostly investigated for episodic memory, given its important role in encoding and retrieval (59–62). Literature has reported increased activation in the hippocampus during episodic memory tasks (63) but reduced functional connectivity of the hippocampus (26, 63) in ASD compared to their non-ASD peers.

In sum, mounting evidence based on behavioral studies implies a profound memory challenge in autistic individuals across different format(s) and types of memory (6, 24, 30), but the neurobiological basis of their memory challenges has not been well characterized quantitatively (9). Therefore, the overarching aim of the current study is to reveal the consistent findings of previous neuroimaging studies on memory functions in ASD. Using a meta-analytic and quantitative approach, specifically, the Activation Likelihood Estimation (ALE) method (64–66), we hope to reveal the brain regions consistently shown in the literature with different activation between ASD and non-ASD. Furthermore, as we have discussed, both behavioral and neuroimaging studies have suggested that the memory challenges in ASD depend on the content (visual vs. verbal) and type of memory (WM vs. episodic memory). Therefore, we also plan to explore whether different brain regions are shown to underlie the memory dysfunctions for different contents and types of memory.

## Method

**Figure 1:**
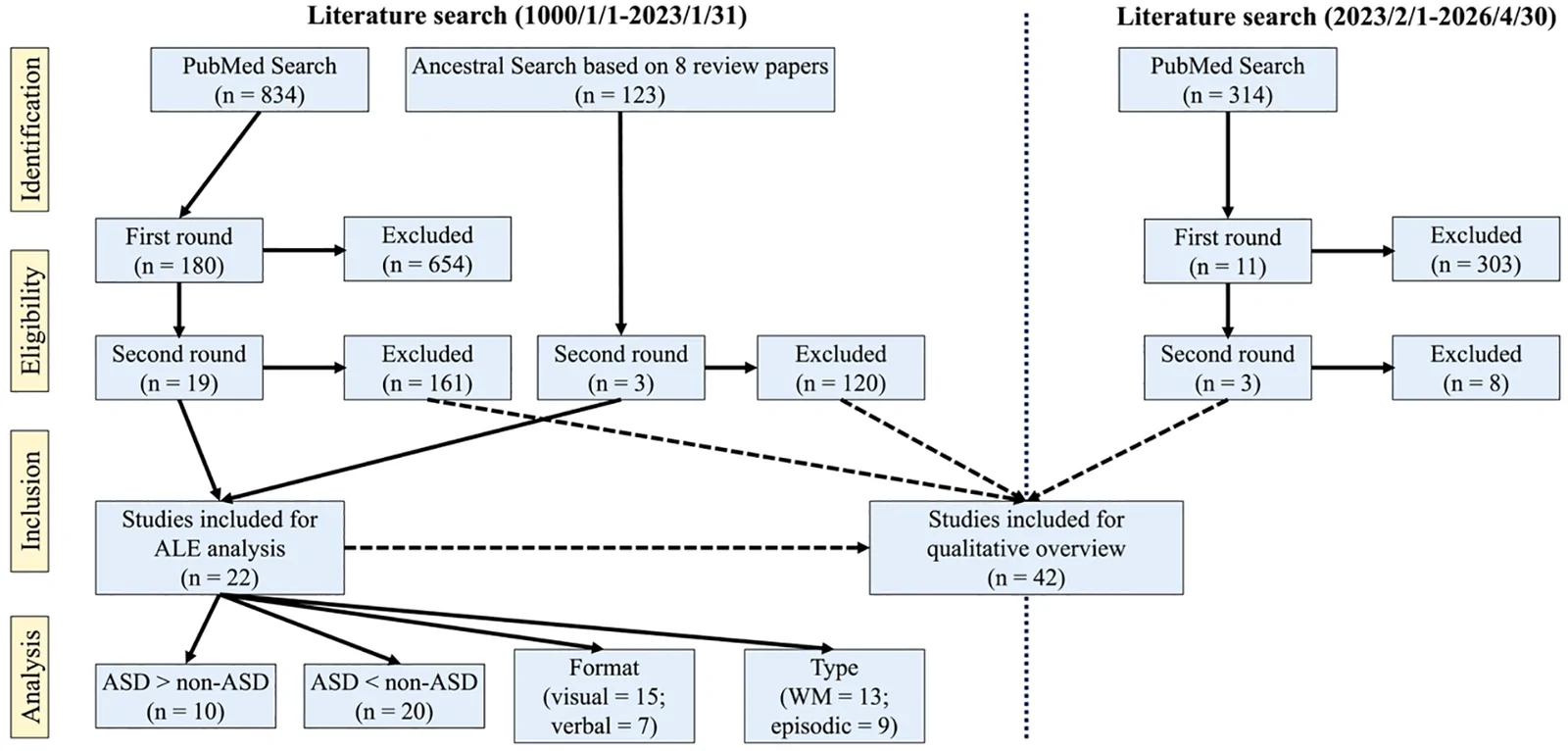
Flowchart of the literature search for the ALE meta-analysis and qualitative overview.

Our study followed the standard procedure of ALE meta-analysis of neuroimaging studies (65), and we also included a flowchart (**Figure 1**) for the literature selection according to the PRISMA guideline (67).

### Study selection

We conducted a comprehensive search and used PubMed as the database. The reason for primarily relying on the PubMed database was that we focused on neuroimaging studies on ASD, and it was most relevant due to its clinical relevance. Firstly, the search terms used in this study were (“brain”[Title/Abstract] OR “imaging”[Title/Abstract] OR “MRI”[Title/Abstract] OR “PET”[Title/Abstract] OR “MEG”[Title/Abstract] OR “neuro”[Title/Abstract]) AND (“autism”[Title/Abstract] OR “Asperger”[Title/Abstract] OR “savant”[Title/Abstract] OR “pervasive developmental disorder”[Title/Abstract]) AND “memory”[Title/Abstract]. This search covered the time range from 1000/1/1 to 2023/1/31, and returned a total of 834 articles.

In the initial round of the screening, we focused on titles and abstracts to select relevant articles on the topic of ASD and memory. All review articles found in this round were labeled for ancestry search later. For primary source research articles, they need to satisfy the following criteria: (a) inclusion of human participants diagnosed with ASD; (b) a memory task or rest to assess the memory ability in ASD; and (c) inclusion of a neuroimaging method (such as MRI, PET, MEG). In this round, all primary reviewers were trained to correctly identify articles based on title and abstract according to the inclusion/exclusion criteria with 20 sample articles. This initial screening resulted in a total of 180 articles.

For the second round of screening, we conducted a more in-depth review of the 180 articles to select articles with the following inclusion criteria: (a) with a memory task or measure (standardized test, working/associative memory tasks/recognition tasks, etc.), (b) with neuroimaging data that were reported within the MNI or Tailarach spaces), and (c) a report of corresponding brain regions. After the second round of filtering, we obtained 19 articles that met the criteria. Then, based on the review articles, we further examined their references to find primary source research articles that may satisfy our inclusion criteria. In this round, all primary reviewers were split into two groups, and each group checked half of the selected articles to classify the eligibility with three options: “Yes”, “No”, “Unclear” (1st reviewer decision). Then, each group reviewed the other half of the selected articles with the same decision options (2nd reviewer decision). The agreement rate (same decision for 1st and 2nd reviewers) was 0.906. The lead researcher reviewed all articles with inconsistent decisions or two “Unclear” decisions from 1st and 2nd reviewers, and made the final decision based on the inclusion/exclusion criteria. After a scrutiny, a total of 3 articles were found via the ancestry search. In total, 22 studies were included in the quantitative ALE analysis of this study.

Among those 22 studies, the following information was extracted: (a) gender and age of the ASD group; (b) the number of participants in the ASD and non-ASD groups; (c) mean IQ of the ASD and non-ASD groups; (d) memory tasks and imaging methods corresponding to the study; (e) diagnostic criteria of ASD; (f) type of neuroimaging data (e.g., task activation, structure differences, functional connectivity; (g) MNI or Tailarach peak coordinates for each brain region; (h) corresponding brain regions; and (i) the direction of the effect (ASD>non-ASD or ASD<non-ASD). Then, based on the task information, each study was classified with its memory content (visual vs. verbal) and memory type (WM vs. episodic).

In order to provide a more comprehensive view of the group differences between ASD and non-ASD, we also collected data from an additional 17 neuroimaging studies that reported a group comparison without specific coordinates or no group differences. Combining the 22 studies used in the ALE analysis and these 17 studies, we planned to examine the proportion of studies showing different directions of the group differences (e.g., ASD < non-ASD, ASD > non-ASD, etc.) and how the differences depended on the format (visual vs. verbal) and the type (WM vs. Episodic). This qualitative analysis could provide us with an overview of the existing findings.

### ALE meta-analysis procedure

All Talairach coordinates were converted into MNI space using the Talairach to MNI (SPM) transform for analytic consistency provided by the GingerALE default tool (version 3.0.2; 64, 65, 68). We also used GingerALE to conduct all the analyses reported here. From the final sample of 22 studies, we conducted a few within-study ALE meta-analytic procedures. The within-study comparison analysis was based on peak coordinates that were reported for the group differences between ASD and non-ASD. The within-study comparison in ALE meta-analysis can reveal the convergence on brain regions in the literature, with a significant difference between ASD and non-ASD. The first within-study ALE meta-analysis was conducted with all coordinates regardless of the direction of the effect. This analysis would reveal brain regions underlying the memory challenges in ASD in a domain-general way, i.e., independent of the direction of the effect, memory content, and memory type. The second within-study ALE meta-analysis would be based on coordinates showing directional effects (ASD > non-ASD or ASD < non-ASD) separately, revealing the consistent hyper- or hypo-effect in brain regions for memory. The last within-study ALE analysis explored further how the consistently reported brain regions for memory functions in ASD depend on the memory content (visual vs. verbal) and memory type (WM vs. episodic) by using the corresponding coordinates separately. The number of studies and coordinates used in different analyses was summarized in **Table 1**.

**Table 1:** Summary of the number of studies and foci included in each ALE meta-analysis.

	All	ASD > non-ASD	ASD < non-ASD	Visual	Verbal	WM	Episodic
No. of Studies	22	10	20	15	7	13	9
No. of Foci	213	80	113	162	47	145	68
No. of Participants	880	400	800	600	399	520	360

We used the same procedure and parameter settings for all analyses. The single-dataset analysis with the corresponding coordinates was carried out first to create the activation maps, including the foci and a Gaussian blur with a full width at half maximum (FWHM). Each study’s sample size was used to determine the FWHM for the foci, with a larger sample size resulting in a larger and tighter curve around the foci peaks. Given the relatively small number of studies included in the ALE analysis, we used an uncorrected p-value of 0.001 and a 100 mm^3^ volume size for thresholding the ALE maps. With a higher threshold (cluster-level FWE correction at 0.05), we found no surviving cluster.

Again, we should emphasize that this threshold is chosen due to the small number of studies, and therefore it is susceptible to Type I errors. To assess the stability of observed clusters at this threshold, we used the Jackknife sensitivity test to reveal whether each cluster can be found when coordinates from one of the 22 studies were excluded. Therefore, the highest stability will be 22/22, suggesting that the cluster was repeatedly found when each of the 22 studies was excluded for the ALE analysis.

### Qualitative analysis of existing literature

The coordinate-based ALE analysis relies on accurately reported loci from neuroimaging studies and could introduce bias in the conclusions. First, if studies find no difference between ASD and non-ASD groups, these null effects are not accounted for. Second, if studies do not report coordinates (e.g., used anatomically defined ROIs, different neuroimaging methods, or simply omission) but show differences between groups, these findings are overlooked in the ALE meta-analysis as well. Therefore, we conducted a qualitative analysis of the reported findings in the literature, regardless of whether coordinates were provided. Based on all the eligible articles, we classified the effect direction of each locus into four categories: ASD < non-ASD, ASD = non-ASD, ASD > non-ASD, and mixed (i.e., reporting more than one direction). Then, we calculated the proportion of each category based on the total number of articles examined. With this approach, we would be able to provide a more comprehensive outlook of the tendency of findings in the literature on the group differences at the cost of no reference to specific brain regions. We believe that this analysis will potentially alleviate bias issues.

### Literature from the more recent studies

Since our original search ended on 2023/01/31, we have conducted another search using the same search terms in PubMed from 2023/02/01-2026/04/30. The search returned 314 articles. The primary reviewer reviewed all articles by title and abstract and decided that 11 could pass the first round of screening. Further examination based on the inclusion/exclusion criteria for the second round yielded only 3 eligible articles. One of them showed no difference between groups (71), and two of them did not report coordinates (72, 73). Therefore, this newer literature will not change our findings for the ALE meta-analysis. We then just added them to the qualitative analysis.

## Results

All studies and gathered data can be found in **Table 2**, and more detailed information on each study is listed in **Table 3**. The whole literature selection process is summarized in **Figure 1**. For studies included in the ALE meta-analysis, we summarized the information on the number of studies, number of loci, and number of participants in **Table 1**.

**Table 2:** Detailed information of the study type and quality of included studies.

First author (year)	Study design	Specific task detail	ASD measures used	Analytical method	Thresholding
Barendse (138)	Cross-sectional	N-back task with images	ADOS	Task activation	corr. p <0.05
Chantiluke (115)	Cross-sectional	N-back with letters	ADOS, ADI-R, ICD-10	Task activation	Peak: p < 0.05; Cluster: p < 0.01
Chien (27)	Cross-sectional	Recognition tasks with visual images	ADOS	Functional connectivity	corr. p <0.05
Cook (116)	Cross-sectional	Association memory test with images	ADOS, ADI-R, BRIEF	Task activation	not specified
Cooper (26)	Cross-sectional	Association memory test with images	AQ	Task activation	corr. p <0.05
Daedelow (53)	Case-control	N-back tasks with numbers	ADOS-2	Task activation	corr. p <0.05
Damarla (69)	Cross-sectional	Target detection with visual stimuli	ADI-G and ADOS	Task activation	uncorr. p < 0.005
Gaigg (36)	Cross-sectional	Word association test	DSM- IV & ADOS	Task activation	p < = 0.005 corr.; voxel size > 10
Greimel (70)	Cross-sectional	Recognition tasks with visual images	DSM IV, ADOS-G, ADI-R	Task activation	peak: uncorr. p <0.001; cluster: corr. p < 0.05
Haigh (117)	Cross-sectional	Spatial and letter span task	ADOS	Brain Structure - White Matter	not specified
Hashimoto (118)	Case-control	Picture-name matching task	ADI-R/ADOS	Functional connectivity	corr. p <0.05
Hawco (119)	Cross-sectional	N-back task with dots	ADOS	Task activation	corr. p <0.05
Hazlett (120)	Cross-sectional	California Verbal Learning Test	DSM-IV	Task activation	not specified
Hogeveen (121)	Cross-sectional	Association memory test with images	ADOS	Task activation	corr. p <0.05
Howard (122)	Cross-sectional	Face recognition task	DSM-IV	Brain Structure - Gray Matter	p <0.05 unspecified
Kana (57)	Cross-sectional	Word recognition task	ADI-R&ADOS	Task activation	p < 0.05, uncorr.; voxel size > 579
Kaufmann (54)	Cross-sectional	Memory span with objects	ADOS, ADI-R, DSM IV	Brain Structure - Gray Matter	not specified
Kleinhans (123)	Cross-sectional	N-back task with images	ADOS& ADI-R	Task activation	not specified
Koshino (56)	Cross-sectional	N-back task with letters	ADI/ADOS	Task activation	t > 5.5; voxel size > 160
Koshino (58)	Cross-sectional	N-back task with faces	ADOS/ADI-R	Task activation	p <0.005 uncorr; voxel size > 10
Laidi (124)	Cross-sectional	Memory recall of images	ADI-R and ADOS	Task activation	not specified
Lu (125)	Cross-sectional	Verbal memory span task	ADOS/ADOS-2)	Brain Structure - White Matter	corr. p <0.05
Luna (126)	Cross-sectional	Spatial location identification	ADI-R/ADOS	Task activation	t > 7 and t > 4
Lynn (127)	Cross-sectional	Recognition tasks with visual images	ADOS, ADI	Functional connectivity, ROI	uncorr. p < 0.05
Morais (71)	Cross-sectional	N-back tasks with numbers	ADOS-2	ROI	not specified
Neumann (55)	Cross-sectional	Word recognition task	ADI-R	Task activation	p < 0.05, not specified
Noonan (128)	Cross-sectional	Word recognition task	DSM-IV, ADI-R	Functional connectivity	corr. p <0.05
O’Brien (129)	Cross-sectional	Recognition tasks with speech stimuli	ADOS	Task activation	corr. p <0.05
Persichetti (72)	Cross-sectional	Recognition tasks with visual images	DSM-V	ROI	not specified
Pierce (130)	Cross-sectional	Recognition tasks with faces	DSM-IV, ADOS	ROI	corr. p <0.05
Rahko (131)	Cross-sectional	N-back task with images	ADI-R & ADOS	Task activation	corr. p <0.05
Silk (132)	Cross-sectional	Memory object rotation task	ASSQ	Task activation	peak: uncorr. p <0.001; cluster: corr. p < 0.05
Tam (133)	Cross-sectional	N-back task with faces	ADOS	Task activation	corr. p <0.05
Trontel (134)	Cross-sectional	Memory tasks with verbal and visual stimuli	ADOS, ADI-R, DSM IV	Brain Structure - Gray Matter	not specified
Unveren (73)	Cross-sectional	Recognition tasks with visual images	ASSQ	Task activation	corr. p <0.05
Urbain (42)	Cross-sectional	N-back task with abstract visual stimuli	ADOS- G	Task activation	corr. p <0.05
Urbain (135)	Cross-sectional	N-back task with abstract visual stimuli	ADOS- G	Functional connectivity	p < 0.005, not specified
Vogan (136)	Cross-sectional	color-matching task	ADOS	Task activation	corr. p <0.05
Vogan (39)	Cross-sectional	Letter matching task	ADOS	Task activation	corr. p <0.05
Vogan (139)	Cross-sectional	N-back tasks with images	ADOS	Task activation	corr. p <0.05
Walsh (137)	Cross-sectional	Memory recall of abstract visual stimuli	ADOS-2 Social affective	Brain Structure - White Matter	uncorr. p < 0.05
Yuk (43)	Cross-sectional	N-back task with abstract visual stimuli	ADOS-G or ADOS-2	Functional connectivity, ROI	corr. p <0.05

ADOS, Autism Diagnostic Observation Schedule; ADI-R, Autism Diagnostic Interview – Revised; DSM, Diagnostic and Statistical Manual of Mental Disorders; ASSQ, Autism Spectrum Screening Questionnaire; AQ, Autism Spectrum Quotient; corr., corrected; uncorr., uncorrected.

**Table 3:** Summary of the studies included in the meta-analysis.

First author (year)	Gender (F:M)*	Age (years)*	N (ASD)	N (NA)	Mean IQ (ASD)	Mean IQ (NA)	Memory task	Imaging method	Direction of effect	Hippo. effect	Notes
Chantiluke (115)	0:17	15.20	17	22	114	111	Ver_WM	fMRI	1		I
Cook (116)	3:09	13.18	12	19	114.17	122.85	Vis_E	fMRI	1		I
Cooper (26)	9:11	30.90	20	20	10.4	10.8	Vis_E	fMRI	3		I
Daedelow (53)	1:00	48.00	1	14	30	31	Vis_WM	fMRI	3		I
Damarla (69)	2:11	19.00	13	13	109.5	111.5	Vis_WM	fMRI	3		I
Gaigg (36)	1:12	35.60	13	12	106.2	110.2	Ver_E	fMRI	3	1	I
Greimel (70)	0:13	15.90	13	13	108	111.5	Vis_E	fMRI	1		I
Hashimoto (118)	12:29	11.20	41	82	102	107	Mixed_E	fMRI	3		I
Hogeveen (121)	12:35	18.40	47	60	105	110	Vis_E	fMRI	4	4	I
Kana (57)	0:15	21.63	15	15	104	114.29	Ver_E	fMRI	1		I
Kaufmann (54)	2:08	14.70	10	10	102.3	109.5	Vis_WM	sMRI	3		I
Kleinhans (123)	N/A	23.50	19	21	106.7	109.1	Vis_WM	fMRI	3		I
Koshino (56)	1:13	25.70	14	14	100.1	109.1	Ver_WM	fMRI	3		I
Koshino (58)	0:11	24.50	11	11	104.5	108.6	Vis_WM	fMRI	3	1	I
Lynn (127)	N/A	17.20	41	43	112.454	111.919	Vis_E	fMRI	1		I
Noonan (128)	0:10	23.00	10	10	96.7	110	Ver_E	fMRI	4		I
O’Brien (129)	5:18	12.20	23	28	110.6	112.8	Ver_WM	fMRI	1		I
Rahko (131)	8:20	14.60	28	22	94.4	NA	Vis_WM	fMRI	3		I
Urbain (42)	4:16	11.25	20	20	108.25	115.95	Vis_WM	MEG	3		I
Vogan (136)	3:16	11.05	19	17	109.42	115.35	Vis_WM	fMRI	1		I
Vogan (39)	5:22	12.56	27	30	105.52	112.27	Ver_WM	fMRI	1		I
Vogan (139)	1:13	10.90	14	15	111.8	118.8	Vis_WM	fMRI	1		I
Barendse (138)	1:12	14.20	13	13	116.7	113.2	Vis_WM	fMRI	2		ND
Chien (27)	4:33	14.84	37	36	104.41	105.47	Vis_E	fMRI	4		NC
Haigh (117)	9:36	23.89	45	20	107.61	104.95	Mixed_WM	sMRI	1		NC
Hawco (120)	6:18	22.50	24	20	116.67	112.16	Vis_WM	fMRI	2		ND
Hazlett (120)	2:15	27.70	17	17	93.2	111.5	Ver_E	PET	1		NC
Howard (122)	0:10	28.05	10	10	NA	NA	Vis_E	sMRI	3	1	NC
Laidi (124)	7:21	31.88	28	31	110	NA	Vis_E	fMRI	2		ND
Lu (125)	8:25	11.30	25	20	108.9	110.1	Ver_WM	sMRI	2		ND
Luna (126)	2:9	32.30	11	6	100.3	104.64	Vis_WM	fMRI	1		NC
Neumann (55)	0:7	21.40	7	7	118.1	114.7	Ver_WM	MEG	3		NC
Pierce (130)	0:8	27.10	8	10	80.3	NA	Vis_E	fMRI	2		ND
Silk (132)	0:7	14.70	7	9	114	NA	Vis_WM	fMRI	3		NC
Tam (133)	3:19	34.10	22	24	106.7	113.9	Vis_WM	fMRI	2		ND
Trontel (134)	0:38	13.20	38	31	106.7	116.3	Vis_E	sMRI	3	2	NC
Urbain (135)	7:13	11.17	17	20	109.94	115.95	Vis_WM	MEG & MRI	3	1	NC
Walsh (137)	4:21	52.60	25	25	110.8	111	Vis_E	sMRI	1	1	NC
Yuk (43)	14:26	27.17	40	39	111.79	114.34	Vis_WM	MEG	2		ND
Newly added from 2023/02/01-2026/04/30											
Persichetti (72)	N/A	19.48	20	19	115.4	119.3	Vis WM	fMRI	3		NC
Unveren (73)	N/A	15-25	4	8	N/A	N/A	Vis E	fNIRS	1		NC
Morais (71)	0:15	22.9	15	15	109.4	116.3	Vis WM	fMRI	2		ND

*Gender ratio and age were only reported for ASD; N/A, not available; NA, non-ASD; Vis, Visual, Ver, Verbal, WM, working memory, E, episodic memory; for direction of the effect, 1 = ASD < non-ASD, 2 = ASD = non-ASD; 3 = mixed (both ASD < non-ASD and ASD > non-ASD), and 4 = ASD > non-ASD. For notes, I, included in the ALE-analysis; ND, no difference; NC, no coordinates reported.

### Atypical functions in ASD compared to non-ASD for memory tasks

**Figure 2:**
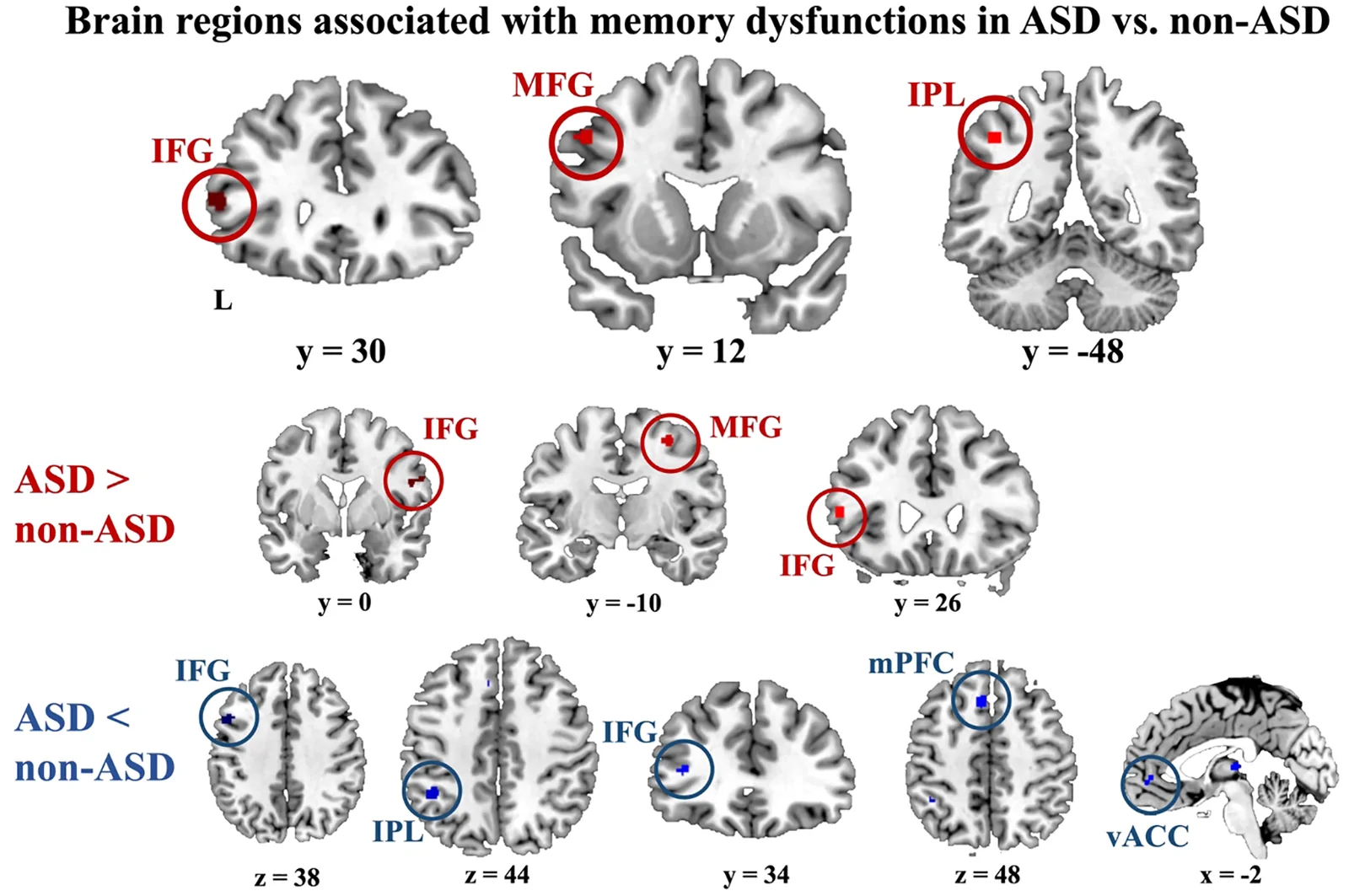
The ALE analysis of the consistent findings on neurobiological correlates for memory challenges in ASD compared to non-ASD. The top row showed the brain regions consistently reported with group differences, and the two bottom rows showed the brain regions with directional effects. IFG, inferior frontal gyrus; MFG, middle frontal gyrus; IPL, inferior parietal lobule; mPFC, medial prefrontal cortex; and vACC, ventral anterior cingulate cortex.

First, we examined the consistent neurobiological findings across all studies. This analysis draws results from all 22 experiments with a total of 880 subjects and 213 foci. We found consistent reports of differences between ASD and non-ASD individuals for the left inferior frontal gyrus (IFG), the left middle frontal gyrus (MFG), the left inferior parietal lobule (IPL), and the thalamus (**Figure 2**, **Table 4**). The sensitivity test showed that all four loci were stable, and they were revealed 20 times out of 22 times when each study was excluded. Although all four loci had the same stability value (20/22), it was not due to the exclusion of the same two studies.

**Table 4:** Brain regions showing consistent dysfunctions for memory in ASD compared to non-ASD.

Cluster	Hemisphere	Region (BA area)	Peak center (x, y, z) in MNI Space	Cluster size (mm^3^)	ALE value	Jackknife sensitivity
All coordinates						
1	Left	IFG (BA 45/46)	-54, 30, 10	432	0.0201	20/22
2	Left	MFG (BA 8/9)	-48, 12, 38	192	0.0155	20/22
3	Left	IPL (BA 40)	-42, -48, 44	160	0.0161	20/22
4		Thalamus	0, -20, 8	120	0.0138	20/22
ASD > non-ASD						
1	Right	IFG/PreCG (BA 9/44)	52, 0, 22	504	0.0110	
2	Right	PreCG/MFG (BA 6)	32, -10, 56	312	0.0136	
3	Left	IFG (BA 45)	-52, 26, 8	264	0.0131	
ASD < non-ASD						
1	Left	MFG (BA 8/9)	-48, 12, 38	352	0.0155	
2	Left	IPL (BA 40)	-42, -48, 44	312	0.0161	
3		Thalamus	0, -20, 8	272	0.0137	
4	Left	Medial PFC (BA 8)	-6, 22, 48	240	0.0133	
5	Left	MFG (BA 46)	-38, 34, 10	144	0.0112	
6	Left	Ventral ACC (BA 32)	-2, 50, -2	112	0.0111	
Visual						
1	Left	MFG (BA 8/9)	-48, 12, 38	312	0.0155	
2	Left	IPL (BA 40)	-42, -48, 44	232	0.0160	
3		Thalamus	0, -20, 6	176	0.0137	
4	Left	Medial PFC (BA 8)	-6, 24, 46	176	0.0131	
5	Right	PreCG/MFG (BA 6)	32, -10, 56	152	0.0136	
Verbal						
1	Left	IFG (BA 45/46)	-54, 30, 10	744	0.0209	
WM						
1	Left	IPL (BA 40)	-42, -48, 44	256	0.0160	
2	Right	PreCG/MFG (BA6)	32, -10, 56	216	0.0136	
3		Thalamus	0, -20, 6	208	0.0137	
4	Left	Medial PFC (BA 8)	-6, 22, 48	200	0.0132	
5	Right	Precuneus (BA 7)	16, -52, 42	152	0.0125	
6	Right	MFG (BA 8)	34, 32, 42	128	0.0120	
Episodic						
1	Left	IFG (BA 45)	-52, 26, 8	304	0.0120	
2	Left	MFG (BA 46)	-38, 34, 10	272	0.0112	
3	Right	MTG (BA 22/37)	48, -42, 2	200	0.0101	

IFG, inferior frontal gyrus; MFG, Middle frontal gyrus; IPL, Inferior parietal lobule; PreCG, Precentral gyrus; PFC, prefrontal cortex; ACC, anterior cingulate cortex; MTG, middle temporal gyrus.

### Hyper- and hypo-functions in ASD compared to non-ASD for memory tasks

We examined consistent neurobiological findings that showed a directional effect, either ASD > non-ASD (i.e., hyper-function) or ASD < non-ASD (i.e., hypo-function). The ASD > non-ASD analysis was based on 10 experiments with a total of 400 subjects and 80 foci, revealing the consistent reports of differences in bilateral prefrontal regions, such as right dorsal and ventral aspects of the precentral gyrus, along with right MFG and IFG, but also left IFG. The ASD < non-ASD analysis was based on 20 experiments with a total of 800 subjects and 133 foci. We found consistent reports of differences between ASD and non-ASD individuals, mostly in the left hemisphere, such as prefrontal regions, i.e., MFG, medial prefrontal cortex (mPFC), as well as IPL and ventral anterior cingulate cortex (vACC; **Figure 2**, **Table 4**).

### Differential effects associated with visual and verbal stimuli

**Figure 3:**
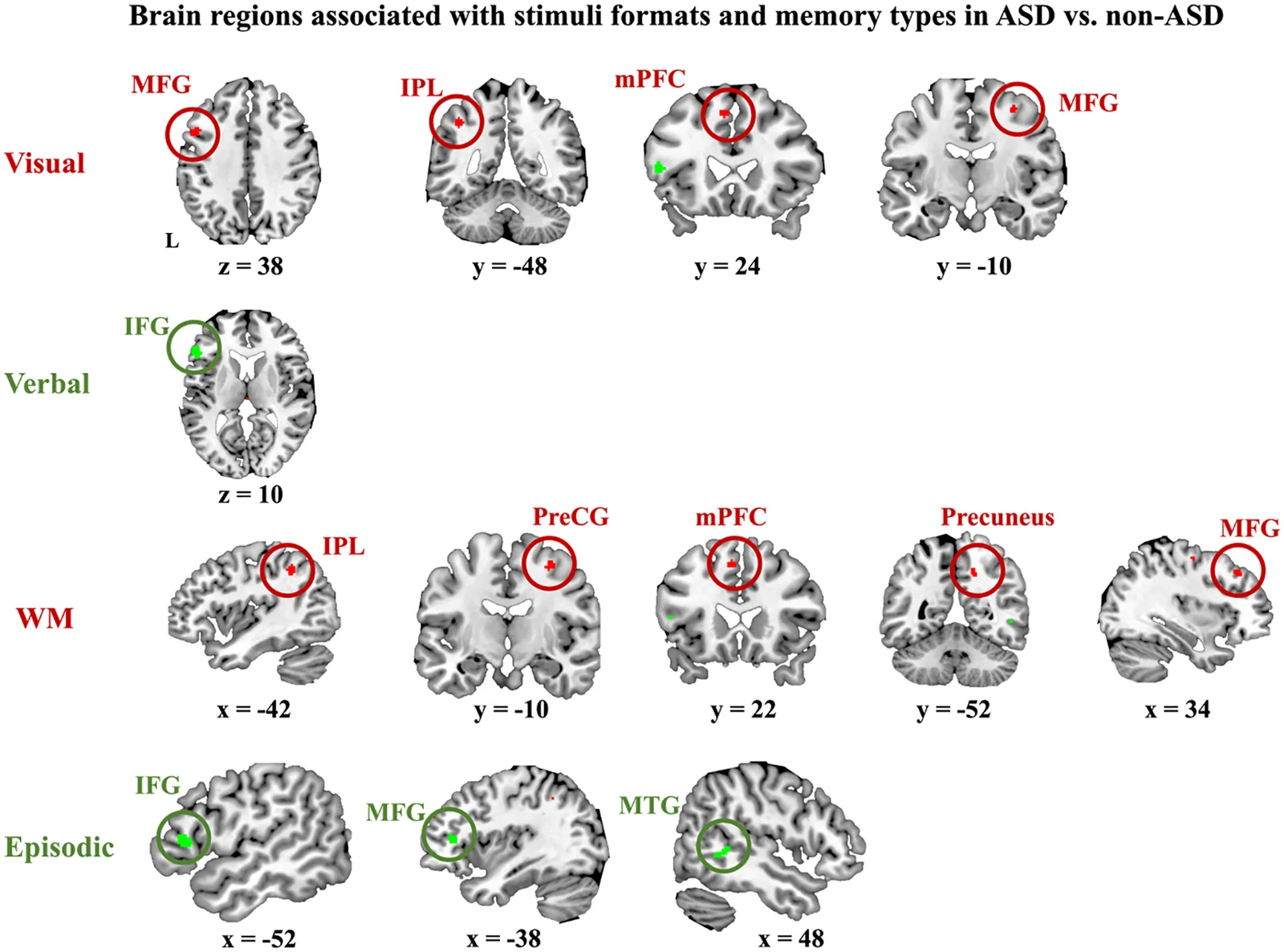
The ALE analysis of the consistently reported brain regions underlying memory challenges in ASD compared to non-ASD for different stimuli formats (visual vs verbal) and memory tasks (working memory vs. episodic memory). IFG, inferior frontal gyrus; MFG, middle frontal gyrus; IPL, inferior parietal lobule; mPFC, medial prefrontal cortex; PreCG, precentral gyrus; MTG, middle temporal gyrus.

To further uncover the neurobiological dysfunctions in ASD, we examined the consistent findings in brain regions for visual and verbal stimuli separately. A total of 15 experiments with 162 foci and 600 participants were included in this analysis for visual stimuli, and a total of 7 experiments with 47 foci and 399 participants were included in the analysis for verbal stimuli. For visual stimuli, the results were very similar to those of the hypo-function analysis. The consistent findings were mostly in the left hemisphere, including frontoparietal regions such as MFG, mPFC, and IPL, and one prefrontal region on the right hemisphere. The results for verbal stimuli only revealed the left IFG (**Figure 3**, **Table 4**).

### Differential effects associated with working memory and episodic memory tasks

To explore the neurobiological underpinnings of different types of memory in ASD, we examined consistent findings during working memory (13 studies with 145 foci from 520 participants) and episodic memory (9 studies with 68 foci from 360 participants) tasks. For working memory tasks, we found consistent effects in a few brain regions in the right hemisphere, such as the MFG, precuneus, and the precentral gyrus. In addition, the left IPL and mPFC seemed to show consistent differences between ASD and non-ASD groups. For the episodic memory task, we did not observe a consistent finding in the hippocampus, but in the IFG, the MFG, and the right middle temporal gyrus (MTG), which is close to the right temporoparietal junction (rTPJ; **Figure 3**, **Table 4**).

### A qualitative overview of the group differences in neuroimaging studies on memory

**Figure 4:**
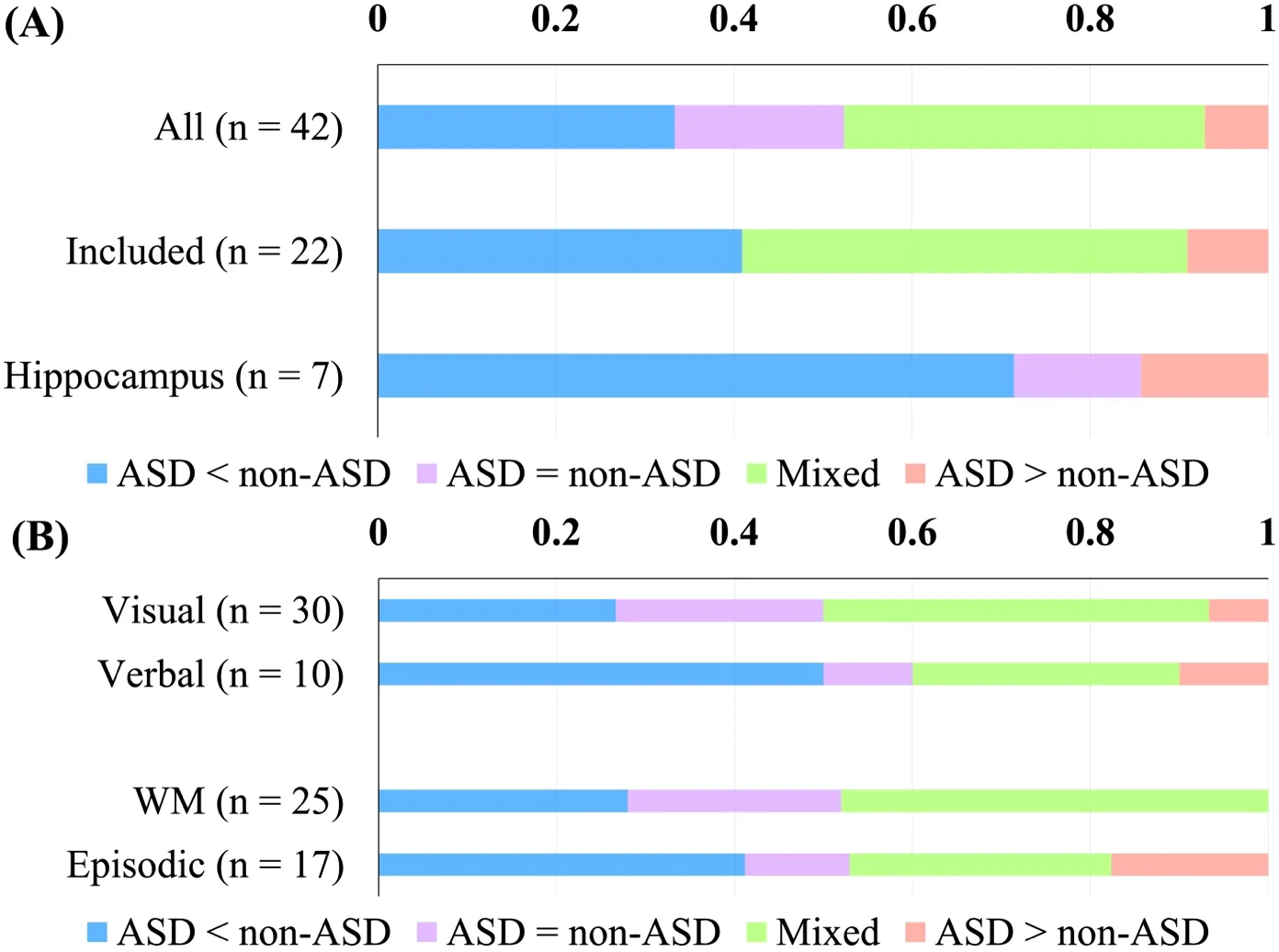
A qualitative overview of neuroimaging studies on memory functions in ASD compared to non-ASD. **(A)** shows the proportion of studies that reported different directional effects in all studies (n = 42), studies included in the ALE analysis (n = 22), and studies reporting effects in the hippocampus (n = 7); **(B)** shows the proportion of studies that reported directional effects using different formats of the stimuli and memory tasks. Mixed = reported both ASD < non-ASD and ASD > non-ASD effects.

In **Figure 4A**, we can see that the findings were very mixed in all the studies we examined (n = 42) or in all the studies included in the ALE analysis (n = 22). However, about 35%~40% of the studies only showed the hypo-functions, i.e., ASD < non-ASD, compared to less than 10% of the studies that only showed the hyper-functions, i.e., ASD > non-ASD. It seems that the autistic individuals were more likely to show deficient functions associated with their memory abilities. Since we did not observe the hippocampus in our ALE analysis, we specifically examined the 7 studies that reported effects for the hippocampus. Interestingly, the majority of the studies (>70%; 5 studies) showed that ASD showed lower functions in the hippocampus compared to non-ASD during memory tasks. We also examined the direction of the effects broken down by the stimulus format (visual vs. verbal) and task type (WM vs. episodic memory) in **Figure 4B**. The effects were again very mixed, but the proportion of studies reporting different effects differed across different conditions, suggesting that the format and task type indeed modulate the observed results. This finding is consistent with the findings from the ALE meta-analysis.

## Discussion

Our ALE meta-analysis with all studies revealed the most consistently reported brain regions associated with memory challenges in ASD in canonical fronto-parietal regions in the left hemisphere for memory functions, such as the IFG and MFG, as well as the IPL. When we examined the brain regions showing directional effects, it was intriguing that hyperfunction in ASD was more consistently found in the right hemisphere, whereas hypofunction in ASD was more consistently found in the left hemisphere, including left prefrontal regions, IPL, and the vACC. Along with our qualitative overview of the literature, this suggests that memory challenges in ASD are likely associated with deficient functions in the brain regions of the left hemisphere, but also some excessive functions in the right hemisphere. Furthermore, both ALE and qualitative analyses pointed out that the underlying brain correlates of atypical memory abilities in ASD depend on the stimulus format (visual vs. verbal) and the memory task (working memory vs. episodic memory). However, it is critical to note that our ALE meta-analysis separated by stimulus format and memory task were conducted with an extremely low number of studies. Therefore, the results from these analyses should be evaluated with caution due to low power and lack of reliability. As a result, our following discussion will focus mostly on the brain regions revealed in the main analysis (i.e., with all studies) and the directional analysis.

Overall, our quantitative meta-analysis confirmed some claims on the hemispheric and anterior-posterior asymmetry in brain dysfunctions underlying the memory challenges in ASD (9). First, consistently, most of the brain regions revealed from our meta-analysis are frontal regions, such as IFG and MFG, and these findings support the idea that the memory challenges in ASD may result from a top-down mechanism, instead of bottom-up mechanisms in low-level sensory processing. Regarding the left-right asymmetry, our meta-analysis (both quantitative ALE analysis and qualitative overview) seems to suggest that the memory challenges in ASD originate from insufficient engagement of critical regions for memory functions in the left hemisphere as well as an over-engagement of counterparts on the right hemisphere, especially in the prefrontal cortex. Our result is contradictory to the previous qualitative analysis on memory dysfunctions in ASD (9), but consistent with other neuroimaging studies examining the hemispheric asymmetry in ASD functional and structural networks (74–78). Specifically, there is a rightward asymmetry in ASD compared to non-ASD in their functional (74) and structural (78) networks, including visual, auditory, motor, executive, attention, and language networks, and the asymmetry is linked to their atypical visual-semantic processing (75), core symptoms like restricted and repetitive disorder (78), and attentional skills (76). Thus, we think that our findings converge with the large body of literature, suggesting that cognitive and social challenges in ASD may stem from the asymmetrical engagement of left and right brain regions. However, given that the memory function in ASD is understudied and our meta-analysis was based on a small sample of studies, future studies should further investigate the hemispheric asymmetry for memory in ASD.

In our ALE meta-analysis, the left IFG was found as one of the most consistent regions. This effect was possibly hypo-function in ASD and driven by the verbal stimuli and episodic memory tasks. The left IFG is commonly associated with verbal memory functions (79, 80) and semantic control (81–84). It has been suggested for processing and social interaction in ASD as well (85, 86). For example, one fMRI study found that abnormal left IFG functional connectivity was responsible for social attention and social language processing deficits in ASD (85). Thus, our analysis supports previous studies on the role of left IFG and highlights its potential role in verbal memory and attention functions in autistic individuals.

Both MFG and IPL are conventionally considered part of a fronto-parietal attention network (87–89), supporting functions such as working memory (90, 91). The findings of our meta-analysis suggest that autistic individuals probably do not engage this network enough during working memory tasks, especially with visual stimuli. Previous studies indeed observed that less activation in the left MFG and IPL during a working memory task may correlate to diminished working memory updating in ASD (54, 58, 69, 70, but also see 53). One fMRI study also found lower activation in the left IPL in ASD during a verbal recollection task (57). This may also suggest the role of IPL, especially, angular gyrus, in the retrieval of semantic and conceptual parts of episodic memory (92–96). Last, the thalamus is thought to be involved in sensory relay (97), and studies have shown that reduced activation in the thalamus could be involved in the increased sensory reactivity in ASD (98). This region is also thought to be involved in working memory (99), and ASD was reported to show reduced functional connectivity in the cortico-striatal-thalamic network during an n-back task (100). Therefore, future studies should examine the role of the thalamus in memory functions in ASD in more detail.

A couple of other regions are worth mentioning based on the ALE meta-analysis in the context of ASD. First, the medial prefrontal cortex was found to show reduced activation during memory tasks in ASD. The mPFC is commonly considered part of the default model network and relates to social functions such as self-perception and emotional regulation (89, 101–103). However, the mPFC cluster we found was more dorsal and closer to the ACC, which has been shown in recognition memory (104), cognitive control (105), and knowledge retrieval (106). Similarly, the ACC is conventionally considered part of the salience network (89, 107), but it is also well-documented for its role in cognitive control and social functioning in autistic individuals (108–110). Therefore, the additional findings of mPFC and ACC outside the fronto-parietal memory network in our meta-analysis imply that memory challenges in ASD are possibly affected by the extended social processing network (57, 111, 112).

Surprisingly, we did not find converging evidence that the hippocampus is underlying the memory challenges, especially for the episodic memory tasks. This finding is inconsistent with the recent findings, which argue for its crucial role in ASD (8). Two potential explanations exist here. First, the literature was not consistent about the atypical functions in ASD. Although behavioral and some neuroimaging studies have suggested a role of the hippocampus for episodic memory functions in ASD (23–26), other studies showed no differences between the ASD and non-ASD groups in terms of performance on item and source memory tasks and the function of the hippocampus (63, 113, 114). Second, the neuroimaging studies on the hippocampus and its role in memory functions in ASD remain scarce. For our meta-analysis, we only identified 7 studies that met our criteria, and only 3 studies reported coordinates for the ALE analysis. Thus, it is entirely possible that our ALE analysis did not have enough power to reveal the hippocampus and its role in memory functions in ASD. Future research should further investigate the hippocampus in ASD, especially during tasks that are known to be challenging for autistic individuals (23, 50), and could engage the hippocampus strongly.

We also extended our original analysis with studies added from 2023/02/01-2026/04/30. However, only 3 studies were eligible for the qualitative analysis. This further highlights the need to understand the neurocognitive basis of memory functions in ASD, which is still understudied. Our qualitative analysis of the studies then provided a more comprehensive picture of the existing literature, which potentially provides a way to alleviate the publication bias. Furthermore, future meta-analytical studies should include more studies, if any, with a sensitive assessment, and pre-registration for transparency and an unbiased approach. Finally, these neuroimaging studies include autistic participants with varying cognitive functions and potential comorbid conditions with other clinical disorders such as ADHD. Thus, future studies should assess how individual factors, such as age, IQ, cognitive abilities, medical and comorbid conditions, could influence the brain functions underlying the memory challenges in ASD.

In conclusion, our study suggests that memory challenges in autistic individuals are primarily associated with decreased function in the left hemisphere, especially the fronto-parietal regions, and an increase in the right frontal regions. These findings potentially suggest an imbalance in the degree of involvement of the different hemispheres of the brain when performing memory tasks. Also, both the format of stimuli (visual and verbal) and the type of memory tasks (WM vs. episodic memory) may influence the activation patterns observed in ASD. However, future studies should investigate the neurobiological dysfunctions for different stimuli and memory tasks in ASD, especially the role of fronto-parietal regions and the hippocampus.

## Data Availability

The original contributions presented in the study are included in the article/supplementary material, further inquiries can be directed to the corresponding author/s.
